# Field transmission intensity of *Schistosoma japonicum* measured by basic reproduction ratio from modified Barbour’s model

**DOI:** 10.1186/1756-3305-6-141

**Published:** 2013-05-16

**Authors:** Shu-Jing Gao, Yu-Ying He, Yu-Jiang Liu, Guo-Jing Yang, Xiao-Nong Zhou

**Affiliations:** 1 National Institute of Parasitic Diseases, Chinese Center for Disease Control and Prevention, Shanghai 200025, China; 2 Key Laboratory of Jiangxi Province for Numerical Simulation and Emulation Techniques, Gannan Normal University, Ganzhou 341000, China; 3 School of Public Health and Primary Care, The Jockey Club Chinese University of Hong Kong, Shatin, Hong Kong; 4 Jiangsu Institute of Parasitic Diseases, Key Laboratory on Control Technology for Parasitic Diseases, Ministry of Health, Wuxi, Jiangsu 214064, China; 5 Key Laboratory of Parasite and Vector Biology, MOH; WHO Collaborating Center for Malaria, Schistosomiasis and Filariasis, Shanghai 200025, China

**Keywords:** *Schistosoma japonicum*, Mathematical model, Prevalence, Basic reproduction ratio, Parameter estimation, Schistosomiasis elimination, People’s Republic of China

## Abstract

**Background:**

Schistosomiasis japonica, caused by infection with *Schistosoma japonicum,* is still recognized as a major public health problem in the Peoples’ Republic of China. Mathematical modelling of schistosomiasis transmission has been undertaken in order to assess and project the effects of various control strategies for elimination of the disease. Seasonal fluctuations in transmission may have the potential to impact on the population dynamics of schistosomiasis, yet no model of *S. japonicum* has considered such effects. In this paper, we characterize the transmission dynamics of *S. japonicum* using a modified version of Barbour’s model to account for seasonal variation (SV), and investigate the effectiveness of the control strategy adopted in Liaonan village of Xingzi county, Jiangxi Province.

**Methods:**

We use mathematical tools for stability analysis of periodic systems and derive expressions for the basic reproduction ratio of *S. japonicum* in humans; we parameterise such expressions with surveillance data to investigate the conditions for persistence or elimination of the disease in the study village. We perform numerical simulations and parametric sensitivity analysis to understand local transmission conditions and compare values of the basic reproductive ratio with and without seasonal fluctuations.

**Results:**

The explicit formula of the basic reproduction ratio for the SV-modified Barbour’s model is derived. Results show that the value of the basic reproduction ratio, *R*_0_, of Liaonan village, Xingzi county is located between 1.064 and 1.066 (very close to 1), for schistosomiasis transmission during 2006 to 2010, after intensification of control efforts.

**Conclusions:**

Our modified version of the Barbour model to account for seasonal fluctuations in transmission has the potential to provide better estimations of infection risk than previous models. Ignoring seasonality tends to underestimate *R*_0_ values albeit only marginally. In the absence of simultaneous *R*_0_ estimations for villages not under control interventions (such villages do not currently exist in China), it is difficult to assess whether control strategies have had a substantial impact on levels of transmission, as the parasite population would still be able to maintain itself at an endemic level, highlighting the difficulties faced by elimination efforts.

## Background

Human schistosomiasis japonica is caused by infection with *Schistosoma japonicum*, a parasitic flatworm (Platyhelminth: Trematoda), and a significant cause of morbidity in oriental Asia, including the People’s Republic of China (P.R. China), the Republic of the Philippines and Indonesia [1]. The disease is a serious threat to human health and contributes to poverty in endemic regions, and has been prevalent in P.R. China for more than two millennia [2]. Schistosomiasis was endemic in 12 provinces of P.R. China, with an estimated 11.6 million people infected as indicated by epidemiological data from the 1950s [3]. Over the last few decades, the prevalence and burden of disease in P.R. China has dropped significantly as a result of sustained efforts and updated intervention strategies within the context of the national schistosomiasis control programme [4,5]. However, the programme is facing new challenges due to the rebound of transmission in some areas, probably influenced by changes in environmental and social factors, particularly in lake regions, which exhibit more ecological complexity than other regions [6]. One of the obvious complexities of *S. japonicum* is that it is a multiple host–parasite system, with many possible non-human, mammal (wild and domestic) reservoirs potentially playing a role in transmission [7]. The results of a nationwide schistosomiasis survey in 2003 revealed that there were still more than 800,000 people infected with *S. japonicum* in P.R. China, with an average infection prevalence of 2.5% [8]. Since then, the national control programme has intensified its efforts, implementing an integrated control strategy aiming to interrupt transmission by focusing on the elimination of infectious sources [9].

The possibility to eliminate schistosomiasis transmission in P.R. China has spurred much debate and discussion on the operational feasibility of such an endeavour. At the same time, mathematical modelling of *S. japonicum* has been crucial to help understand its transmission dynamics and the effects on such dynamics of the interventions implemented [10-14]. Further investigations on the threshold of schistosomiasis transmission, measured by the basic reproduction ratio (*R*_0_) have been undertaken to understand the feasibility of, and effort involved in the shifting of the current strategy from transmission and morbidity control to elimination of the infection reservoir [15,16].

The concept of the basic reproduction ratio can be traced back to the work of Alfred Lotka, Ronald Ross, and others [17-20], but its first application in modern epidemiology was by George MacDonald in 1952, who constructed population models of the spread of malaria [21]. It is often denoted by *R*_0_ and has received much attention in mathematical epidemiology [22-27]. The basic reproduction ratio is typically defined in the epidemiology literature as the average number of secondary cases resulting from a single infected primary case, introduced into a completely susceptible population, during the entire infectious period [28]. This threshold criterion states that when *R*_0_< 1, each infected individual produces, on average, less than one new infected individual, and the infection would be cleared from the population. If *R*_0_> 1, the infection will become endemic in the population providing that susceptibles are replenished. In an endemic infection, we can determine which control measures, and at what magnitude, would be most effective in reducing *R*_0_ below one, providing important guidance for public health initiatives [29].

Mathematical modeling of schistosomiasis transmission, which began with the work of MacDonald in 1965 [30], has been undertaken extensively over the years, both from a theoretical standpoint [31-35], and from the perspective of developing operational research tools for evaluation of control programmes [36-38]. By simulating control interventions, models are used to make theoretical predictions and/or projections about the future trends of schistosomiasis.

Broadly, mathematical models for schistosomiasis can be divided into those describing the intensity of infection (the worm burden per host), such as the early frameworks of MacDonald (1965) and others [28,34,39,40], and those describing the prevalence of infection [32,41-44]. In particular, Barbour (1996), to which we refer here as the Barbour’s model, compares the performance of both approaches for the calculation of *R*_0_ and concludes that intensity of infection frameworks which include density dependence in the snail component alone may lead to its underestimation. The interactions between definitive hosts and snail intermediate host in multihost–parasite systems are more complex than that has been described in Barbour’s model [41] (Table 1). In model (1.1), *a* and *b* are composite parameters. Parameter *a*, involving much biology and sociology, represents the rate at which a single definitive host becomes infected at unit density of infected snails and is difficult to estimate. Parameter *b* is the rate at which snails become infected. For each definitive host, Barbour [41] indicates that the force of infection acting upon a single definitive host species is equal to *aΔy*, where Δ is the density of snails, and *y* is the prevalence of infected snails (Table 2). Barbour [41] defines *R*_0_′ = *abΣ*/(*gμ*) as the basic reproduction ratio for the autonomous dynamical system (1.1), where Σ is the density of hosts, *g* the recovery rate for definitive host infections, and *μ* the per capita mortality rate of snails. It is shown that if *R*^′^_0_ < 1, the only equilibrium is $\bar{P}=\bar{y}=0$, with $\bar{P}$ and $\bar{y}$ denoting, respectively, the average prevalence of infection in hosts and snails, and no endemic infection is possible. If *R*^′^_0_ > 1, infection leads to an endemic steady state. An extended Barbour’s two-host model system that was also described by Barbour 1996 for *S. japonicum,* was used to study human–bovine transmission of schistosomiasis in Jiangxi Province, P.R. China [14]. The analysis showed that treatment of humans that eliminates a fraction of adult worms according to drug efficacy and coverage has an immediate and large effect on human prevalence of infection, whereas treatment or vaccination of bovines with an anti-*S japonicum* fecundity vaccine of imperfect efficacy impacts on human incidence in the longer term [2,45].

**Table 1:** **Interpretation of model (1.1) from Barbour (1996)**[41]

**Code**	**Model**
(1.1)	$$\left\{\begin{array}{l} \frac{\mathrm{dP}}{\mathrm{dt}}=\mathrm{a\Delta y}\left(1-P\right)-\mathrm{gP},\\ \frac{\mathrm{dy}}{\mathrm{dt}}=b\left(\frac{\Sigma }{\Delta }\right)P(1-y)-\mu y, \end{array}\right)$$ The dynamics of schistosome transmission is represented by this set of coupled differential equations
**Parameters**	**Interpretation**
*P*	the prevalence of infection in the definitive host population
*y*	the proportion of infected snails
*a*	the rate of incidence for a single definitive host at unit density of infected snails
*b*	the rate at which an infected definitive host causes snail infections
*g*	the recovery rate for definitive host infections
∆	the density of snails
Σ	the density of definitive hosts
*μ*	per capita death rate of infected snails

**Table 2:** **Model of force of infection with *a *and *b *as composite parameters**

**Code**	**Model**
(From 1.1)	*a* = {rate of a host contacting contaminated water per unit time} × { density of cercariae} × {probability that a cercarial encounter leads to the establishment of an adult parasite}
(From 1.1)	*b* = {rate of egg–laying} × {probability of an egg developing into a miracidium} × {probability that a miracidium penetrates a snail} × {probability that a miracidial penetration into an uninfected snail develops into patent infection}

In the aforementioned framework, the coefficients of the Barbour’s model and its extensions are reasonably simplified. Those coefficients are considered as constants, which are approximated by average values. Our field observations indicate, by contrast, that infection of definitive hosts occurs at different rates every month of a year in the endemic areas, with a high re-infection rate [46]. In fact, natural factors, such as seasonal changes in moisture and temperature, affect the abundance and activity of the intermediate snail host, *Oncomelania hupensis*, and the transmission dynamics of schistosomiasis are in a constant state of flux [47]. Moreover, there are many social factors related to human behaviours accounting for the change of schistosomiasis incidence, such as marked changes of contact rates caused by daily production activities [48]. Therefore, periodic fluctuations in schistosomiasis transmission occur and vary according to local settings, yet such fluctuations have not yet been incorporated into mathematical models of schistosomiasis japonica.

The objectives of the present study are, therefore, (i) to modify the prevalence framework presented by Barbour by incorporating seasonally fluctuating dynamics, (ii) to derive expressions for the basic reproduction ratio with seasonality and compare them with those ignoring seasonality, (iii) to parameterize the model with surveillance data from a study village in Liaonan in Xingzi county, Jiangxi Province, and (iv) to discuss the impact of the interventions implemented on the stability and persistence of *S. japonicum* in the study area.

## Methods

To incorporate seasonal variation with multiple factors into mathematical models, a reasonable method is to assume that some parameters of the model behave as periodic functions [3,49]. In order to overcome the shortages of Barbour’s model as discussed above, we modify the mathematical model for *S. japonicum* transmission by including periodic coefficients. Taking the rates of change with respect to time of infected humans and of infected snails as our variables of interest (thus ignoring non-human definitive hosts), the following assumptions are made: (i) the human population is considered a constant closed system in which it is taken that the death rate is equal to the birth rate and is much smaller than the recovery rate, and hence it is ignored in the corresponding equation, (ii) a snail, whether infected once or several times, releases cercariae at the same rate, (iii) the infectivity of an infected definitive host is not influenced by the number of subsequent infections or by the current parasite burden of such a host. Under these assumptions our aim is to assess the effect of the new integrated control strategy, which has been implemented since 2005, in terms of temporal trends in the magnitude of schistosomiasis transmission measured by *R*_*0*_, based on surveillance data collected at our study site.

### Mathematical formulation

The incidence of schistosomiasis in the real world tends to vary due to seasonal ecological fluctuations. With the aim of assessing the effect of periodic fluctuations on transmission of schistosomiasis, we modify the transmission coefficients, *a* and *b* of Barbour’s framework to reflect seasonality, namely they become functions of time rather than fixed constants, *a*(*t*) and *b*(*t*). Our mathematical model is given by the following equations, with *P* denoting the prevalence of infection in humans and *y* the prevalence of infection in snails (and the remaining parameters as defined above and in Table 1) to keep consistency with Barbour’s model,

(2.1) $$\left\{\begin{array}{l} \frac{\mathrm{dP}}{\mathrm{dt}}=a\left(t\right)\mathrm{\Delta y}(1-P)-\mathrm{gP},\\ \frac{\mathrm{dy}}{\mathrm{dt}}=b\left(t\right)(\frac{\Sigma }{\Delta })P(1-y)-\mu y. \end{array}\right)$$

Here, the incidence rate at which a single definitive host acquires infection at unit density of infected snails, *a*(*t*), and the rate at which an infected definitive host causes snail infections, *b*(*t*), are periodic positive continuous functions of *t* with period *ω* = 365 (days). System (2.1) is called the seasonal variation (SV)-modified Barbour’s model. In this study, we choose the composite functions as follows,

(2.2) $$\left\{\begin{array}{l} a\left(t\right)=a_{0}\left|\;\sin\;\left(\mathrm{\pi t}/365\right)\right|,\\ b\left(t\right)=b_{0}\left|\;\sin\;\left(\mathrm{\pi t}/365\right)\right|, \end{array}\right)$$

with $a=\frac{1}{365}\int_{0}^{365}a\left(t\right)\mathrm{dt}$ and $b=\frac{1}{365}\int_{0}^{365}b\left(t\right)\mathrm{dt}$. Thus, $a_{0}=\frac{\pi }{2}a$, $b_{0}=\frac{\pi }{2}b$, and $a\left(t\right)=\frac{\pi }{2}a|\sin\frac{\mathrm{\pi t}}{365}|$, $b\left(t\right)=\frac{\pi }{2}b|\sin\frac{\mathrm{\pi t}}{365}|$.

### *The basic reproduction ratio* (*R*_0_)

For the SV-modified Barbour’s model, we use operator theory in functional analysis and the monodromy matrix of linear periodic system theory to derive a biologically meaningful threshold index, the basic reproduction ratio *R*_0_. The method, which is motivated by the work of Wang and Zhao [50], presents the *R*_0_ formulation for the SV-modified Barbour’s model first. Numerical computation of the basic reproduction ratio was then conducted using the mathematical programming language MATLAB 7.1.

### Study area and estimation of model parameters

Liaonan village in Xingzi county, Jiangxi province, was selected as our study area. This village had implemented an integrated control strategy, with emphasis on infection source control, since 2005. Calibration of the model was based on parameters from the literature and preliminary analysis of the data collected. The data were obtained from the annual report surveillance data, and included data from 2003 to 2010. Therefore, data from 2003 to 2005 represent a period prior to implementation of the integrated control strategy, with data from 2006 to 2010 representing the period of intensified intervention. The prevalence data of human, bovine and snail were estimated based on the routine surveillance [51]. In the routine surveillance, the residents were screened by antibody-based indirect hemagglutination assay (IHA) and then the positives with dilution titers over 1:10 in IHA were confirmed by Kato-Katz stool examination [52,53]. Infected bovines were detected by the faecal hatching test, briefly, bovine faecal samples (50 g per cattle) were stirred and precipitated with clean water for several times, water was added to the supernatant in a flask and transferred to an incubator maintained at 25±3°C for observation after 1–3 hours to check if any miracidium had hatched in the top surface of water in the flask [54]. The snail intermediate host, *O. hupensis*, was detected on the marshland near the study village connecting to the Poyang Lake, using the methods of randomized sampling with a sampling iron-frame (0.1 m^2^). All of snails inside each sampling iron-frame were collected and dissected under a microscope to check whether the snails were infected with cercaria of *S. japonicum*[55]. The infection rate of humans or snails was calculated based on how many infected residents or infected snails there were out of the numbers examined [56]. The densities of human and bovines were collected from census data in human and bovines, respectively, and snail density was estimated based on the average number of snails in each sampling iron-frame.

For models (1.1) and (2.1), composite parameters *a* and *b*, and composite functions, *a*(*t*) and *b*(*t*) are difficult to measure directly, as they involve contact rates between hosts and snails and the probability of the infection establishing successfully in humans and snails. The difficulty in estimating these composite parameters for schistosomiasis lies in the large amount of unobserved data inherent in the disease process. Here, we use the approach of Wu [57] to define composite parameters as annual average values. Expressions for the functions *a*(*t*) and *b*(*t*) are difficult to determine from reported data, so in Eqn (2.1) they are represented by periodic functions to signify seasonal variation. In our study, optimized expressions of *a*(*t*) and *b*(*t*) are obtained by using the trigonometric functions shown above, with a period 365 days. The values of parameters *a* and *b* are equivalent to the average values of functions *a*(*t*) and *b*(*t*) in one year (365 days), respectively.

The reciprocal of the recovery rate, 1/*g*, is equivalent to the average duration of infection in human hosts. The death rate of infected snails was estimated as the reciprocal of the snails’ life expectancy [57]. The life expectancy of infected snails and the duration of infection of humans were estimated based on data from [14]. It was assumed that the average duration of infection in humans is 4 years. Snail infections were assumed to last an average of 6 months and since snails do not recover from infection, this was assumed to be the life expectancy of infected snails. Thus, we have *g* = 1/4 per year = 1/(4 × 365) per day= 0.00068 per day, and *μ* = 1/6 per month = 1/(6 × 30) per day= 0.0055 per day.

The density of snails, *Δ*(/*m*^2^), and the density of definitive hosts, *Σ*(/*m*^2^), were estimated from annual report surveillance data (Table 3). The surveillance data was collected in July or August each year, rather than monthly, this was one month after mass drug administration with praziquantel for the residents. This yearly data is often used as an annual average value (therefore masking any monthly fluctuations that may have occurred). Therefore, for the purpose of modelling (1.1), it is assumed that schistosomiasis transmission remains in a steady state throughout the year in endemic areas.

**Table 3:** **The data for**$\Delta ,\;\Sigma ,\;\bar{P}$**, and**$\bar{y}$**from annual report data in Liaonan village, Xingzi county, Jiangxi Province, P. R. China**

**Parameter**	**2003**	**2004**	**2005**	**2006**	**2007**	**2008**	**2009**	**2010**
*Δ*(/*m*^2^)	28.0	33.9	91.5	133.5	41.1	17.5	11.3	2.4
*Σ*(/*m*^2^)	0.030	0.020	0.048	0.017	0.031	0.018	0.031	0.018
$\bar{P}$ (%)	0.0671	0.0533	0.0631	0.0454	0.0454	0.0454	0.0453	0.0457
$\bar{y}$ (%)	0.00021	0.00005	0.00013	0.00014	0.00018	0.00050	0.00077	0.00002

Note: *Δ* means the density of snails, *Σ* means the density of definitive hosts, $\bar{P}$ means the average prevalence of infection in hosts in one year, $\bar{y}$ means the average prevalence of infection in snails in one year.

By setting equations of the model (1.1) to zero we find steady-state (equilibrium) values for the prevalence of infection in humans and snails,

(2.3) $$\bar{P}=\left(1-\frac{1}{{R^{\prime }}_{0}}\right)/\left(1+\frac{g}{\mathrm{a\Delta}}\right),$$

(2.4) $$\bar{y}=\left(1-\frac{1}{{R^{\prime }}_{0}}\right)/\left(1+\frac{\mu \Delta }{\mathrm{b\Sigma}}\right).$$

It is assumed that $\bar{y}$ is the equilibrium infected snail prevalence and $\bar{P}$ is the equilibrium prevalence of infection in humans for each year.

## Results

### Calculation of *R*_0_

The mathematical details of the derivation of expressions for the basic reproduction ratio can be found in the Additional file 1: Derivation of *R*_0_ and the proof of the main results.

### Prevalence of infection in humans and snails

The values for the equilibrium infected snail prevalence and the equilibrium prevalence of infection in humans are shown in Table 3. Combining these with the steady-state values of the model, composite parameters *a* and *b* of model (1.1) can be derived from equations (2.3) and (2.4) and are shown in Table 4.

**Table 4:** **The calculated values of composite parameters *a *and *b *from Model 1.1**

**Parameter**	**2003**	**2004**	**2005**	**2006**	**2007**	**2008**	**2009**	**2010**
*a*	0.0085	0.0226	0.0038	0.0017	0.0044	0.0037	0.0037	0.0088
*b*	0.0157	0.0088	0.0224	0.1344	0.0287	0.0598	0.0341	0.0254

Note: *a* refers the infection rate at which an infected snail makes an susceptible definitive host to be infected per unit time (1 day) and per square meter waters, *b* refers the rate at which an infected definitive host causes the snail population to be infected per unit time (1 day) and per square meter waters.

### Numerical simulation and sensitivity analysis

**Figure 1:**
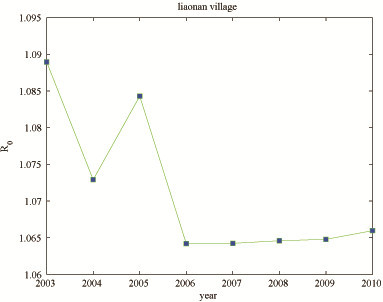
Changes in the basic reproduction ratio in the Liaonan village from 2003 to 2010.

Based on the annual report data in Xingzi county during 2003 to 2010, where a control strategy which focused on control of the infection source was implemented in 2005 [58,59], we investigated the effectiveness of the strategies adopted in Liaonan village of Xingzi county. Table 5 gives the results from the calculation of the basic reproduction ratios for each year comparing the values obtained by using model 1.1 (denoted *R*^′^_0_) with those obtained with model 2.1 (denoted *R*_0_). The values of *R*_0_ are consistently (but not markedly) higher than those of *R*^′^_0_. The values of *R*_0_ are located in the range from 1.064 to 1.089 for schistosomiasis transmission from 2003 to 2010 in the Liaonan village of Xingzi county, which are close to 1 (Figure 1). The values of *R*^′^_0_ obtained with the non-SV model lay between 1.020 and 1.081.

**Table 5:** Numerical results for the annual basic reproduction ratio values according to Models 1.1 and 2.1

**Basic reproduction ratio**	**2003**	**2004**	**2005**	**2006**	**2007**	**2008**	**2009**	**2010**
*R*^′^_0_	1.0705	1.0582	1.0811	1.0202	1.0433	1.0531	1.0424	1.0519
*R*_0_	1.0890	1.0729	1.0843	1.0642	1.0642	1.0646	1.0648	1.0660

Note: *R*^′^_0_ corresponds to the original Barbour’s model (1.1), *R*_0_corresponds to the seasonal variation (SV)-modified Barbour’s model (2.1).

**Figure 2:**
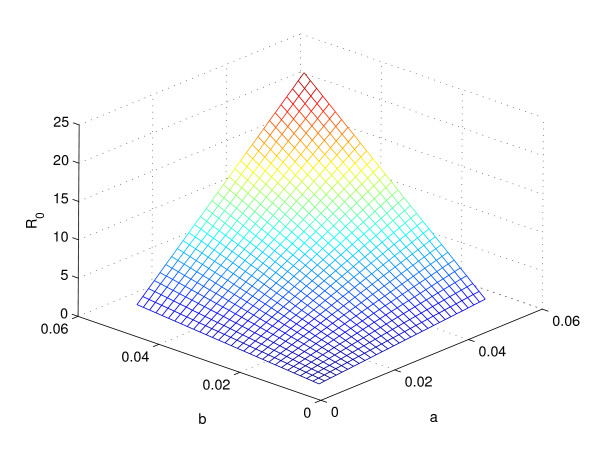
**The relationship between *a*, *b *and *R***_**0**_**.** The graph demonstrates the sensitivity of the basic reproduction ratio to the changes of composite parameters *a* and *b.*

Furthermore, the sensitivity analysis on parameters *a* and *b,*$a=\frac{2}{\pi }a_{0},\;b=\frac{2}{\pi }b_{0}$ and parameters *a*_0_ and *b*_0_ are related to the magnitudes of the seasonal fluctuation *a*(*t*) and *b*(*t*), respectively, showing that parameters, *a* and *b* vary with the basic reproduction ratio. It is also shown that *R*_0_ is linearly related to both *a* and *b* with the pattern that *R*_0_ decreases to a relatively small value (less than 1) only when *a* and *b* are very small (Figure 2).

## Discussion

Recently, much attention has been given to developing schistosomiasis transmission models that can be used to assess and project the effects of control measures. A biologically-motivated model of *S. japonicum* transmission has been described in detail that incorporates human hosts, adult parasites, uninfected and infected snails, free-living miracidia, and free-living cercariae [60]. A mathematical model for the transmission dynamics of *S. mansoni* was also presented, in which the dynamics of miracidia and cercariae were incorporated [16]. The model was analyzed to gain insights into the qualitative features of endemic equilibrium and to allow for determination of the basic reproduction ratio (using the spectral radius of an appropriate matrix as described by Van den Driessche *et al.*[61]). In another study, thresholds for the survival of schistosomes were established after more than two human habitats sharing the same contaminated water resource were observed [15], then control strategies were discussed in the light of these thresholds [15]. Many of these studies, however, have not yet solved the problems associated with estimating *R*_0_, which is remarkably difficult, particularly for vector-borne and indirectly-transmitted diseases, based on field surveillance data [62,63].

In general, the difficulty lies in the large number of parameters necessary to obtain or estimate the basic reproduction ratio. So decisions as to which parameters are the most important are crucial. In this study, we modified the framework presented by Barbour in 1996, adapted for *S. japonicum* transmission, by incorporating periodic functions into those parameters that include the contact rates, to evaluate the effect of control measures in one village in Xingzi county. Parameters *g* (the recovery rate from infection in the humans), *μ* (the mortality rate of snails), Δ (the density of snails per unit water area), and Σ (the density of definitive hosts), can be directly obtained from the reported data. However, there is no information available for the values of parameters *a*(*t*) and *b*(*t*), the transmission coefficients from snails to humans and from humans to snails, respectively, in model (2.1). For simplicity, we employed trigonometric functions for the expressions of *a*(*t*) and *b*(*t*). While detailed information is still lacking, there is little doubt that the composite parameters change over time. The method we used to estimate the composite parameters of model (2.1) in this paper takes into consideration realistic features of the disease and is preferable to the constant coefficients used in other models. However, although our values of the basic reproduction ratio obtained with the SV-model were consistently higher than those obtained with the non-SV model, the difference was only marginal.

Often, the basic reproduction ratio is a useful indicator of both the risk of an epidemic and the effort required to control an infection. Here, we define the basic reproduction ratio *R*_0_ of the SV-modified Barbour's model (2.1) and confirm that the disease would be controlled if *R*_0_ < 1 or sustained at an endemic level if *R*_0_ > 1. On the basis of the surveillance data collected, we observed that the basic reproduction ratio of *S. japonicum* in Liaonan village under control lies in the range of 1.064 to 1.066 after 2005, with slight year-to-year fluctuations. It is shown that although Liaonan village has achieved good results by implementing integrated control strategies, with emphasis on infection source control, the results are not very different from those between 2003 and 2005, before the intensification of control efforts, suggesting that the infection is still able to maintain itself at a low, yet quasi-steady state equilibrium. Other modelling studies have shown that after repeated treatment, and conditioned to non-elimination, the prevalence distribution, in those villages where infection persists at a very low level, it reaches a stationary (termed a quasi-stationary) distribution [31,64-67]. Modelling studies for lymphatic filariasis have also shown that in the vicinity of transmission breakpoints, parasite resilience and the specificities of the host-parasite system and distribution among hosts may make elimination hard to achieve [68].

The composite parameters in our SV-modified Barbour’s model are naturally subject to fluctuation in time, that is, the SV-modified Barbour model is non-autonomous. It should be more realistic than the autonomous system, as seasonal variation in the dynamics of snails and humans occur in the field. Moreover, from our results of numerical computation, we can see that transmission elimination policies, based on computation of the basic reproduction ratio using time-averaged transmission coefficients, may underestimate (albeit slightly) the infectious risk inherent to periodic disease transmission.

Note that the values computed for the basic reproduction ratio depend on model parameters, which can be influenced by the control strategies implemented in the study areas. For better control and elimination of schistosomiasis japonica, it is necessary to obtain a better knowledge of the relationship between control strategies and model parameters, particularly those composite parameters that include the contact rates. The main interventions for schistosomiasis include preventive chemotherapy using praziquantel which impacts on the duration of infection, health education that affects contact rates, mollusciciding which decreases snail survival, environmental management that influences exposure, and sanitation improvement which decreases contamination rates of water sites. On the whole, elimination of schistosomiasis is based on protection of water sources, limitation of water contact, and eradication of intermediate snail hosts [69]. Treatment with praziquantel, which reduces egg output, should reduce contamination rates and impact on *b* (the rate at which humans infect snails). However, it has also been suggested that praziquantel treatment, by killing adult worms and releasing antigens otherwise not seen by the immune system [70], can have a ‘vaccination’ effect by these antigens eliciting some protective immunity (thus impacting on *a*) [71]. The frequency of contact with infected water by humans in the lake and embankment areas can be high. Health education can reduce the frequency of contact with possibly contaminated water, also impacting on *a*. Sanitation improvement should reduce the probability of faeces reaching water sites and therefore the probability of an egg developing into a miracidium, also decreasing *b*. However, sanitation improvement may also reduce exposure and hence *a*. It is well known that reducing the number of snails with extensive mollusciciding, and modifying snail habitats by draining rivers and ditches will reduce contact rates. Chemical mollusciciding in the study village was generally carried out annually. But snails re-appeared in certain environments still suitable for snail breeding. Thus, environmental modification was also carried out in an effort to control transmission more effectively [72].

## Conclusions

### Limitations of our study

The SV-modified Barbour's model for schistosomiasis transmission proposed here has the following limitations. Firstly, in fact, *S. japonicum* is a zoonosis with over 40 species of mammals acting as possible reservoir hosts as stated earlier [73,74]. Bovines are thought to be particularly important for harbouring and transmitting *S. japonicum* in lake and marshland regions of southern China [74-77]. Diagnosis and control of bovine schistosomiasis is vitally important for reducing the prevalence of the disease [14]. Thus, it will be important to be able to include transmission from non-human definitive hosts into the model for evaluation of the efficacy of control programmes in many other disease-endemic areas. Our present model attempts to incorporate seasonal fluctuations of transmission, but in the future we will extend Barbour's two-host model to investigate a multihost model with seasonal fluctuations. Secondly, the basic reproduction ratio completely determines the long-term behaviour of the disease only in theory. We computed yearly basic reproduction ratios according to the expressions derived from our model and the previous non-SV model. Although indicative, this is not sufficient; formulation of stochastic models that allow the feasibility of elimination to be explored, and analysis of empirical studies in near elimination scenarios to determine operational thresholds for elimination will also be required [78]. Thirdly, as transmission rates vary according to time and circumstances, it is difficult to obtain accurate estimates of time-varying composite parameters due to lack of data [79]. In this paper, trigonometric functions with a period of 365 days were proposed to simulate these composite functions. However, more accurate estimation of the composite functions will be important for describing and analyzing the behaviour of the model. Thus the model will be combined with time series data in the future [80].

Actually, a defensible set of parameter estimates is difficult to determine because the intensity of control strategies differentially affects model parameters. The credibility of our conclusions relating to control strategies is weakened by only using annual report data. However, the collected data under trial conditions with relatively stable intervention measures can help us to assess the effectiveness of control strategies. The evaluation of a control strategy usually involves a group with intervention and a group without intervention, yet there are no villages in P.R. China under no intervention. However, in our study village, the fact that our estimates before (2003–2005) and after (2006–2010) intensification of control intervention do not reveal a substantial trend, indicates that estimation of the basic reproduction ratio alone may be insufficient to assess the impact of control strategies effectively.

Finally, we assumed that the study village was a closed system, whilst the issue of connectivity between villages either by movement of hosts or snails (the latter through hydrological connectivity) is central to the discussion of control strategies in low transmission settings in P.R. China [81]. Feng *et al.* (2005) considered this issue in the literature [15], but the models proposed are complex and difficult to parameterise. However, there is no doubt that a metapopulation approach for the understanding of disease persistence in P.R. China will shed light on the landscape of schistosomiasis japonica elimination efforts.

## Competing interests

The authors declare that they have no competing interests.

## Authors’ contributions

SJG, YYH, YJL and XNZ were involved in all study processes including design, data acquisition, analysis and interpretation of the results as well as the drafting of the manuscript. SJG, GJY and XNZ initial study concept and revised all drafts of the manuscript. All authors read and approved the final version of the manuscript.

## Supplementary Material

Additional file 1 **Derivation of *R***_**0 **_**and the proof of the main result.** Click here for file
