# Supplementary material for: Field transmission intensity of Schistosoma japonicum measured by basic reproduction ratio from modified Barbour’s model
Source: Parasit Vectors. 2013 May 16;6:141. doi: 10.1186/1756-3305-6-141 (PMC3667069; doi:10.1186/1756-3305-6-141)
Supplement: Additional file 1 — Derivation of R0 and the proof of the main result. [file 1756-3305-6-141-S1.doc]

**Supplementary Text: Derivation of $R_{0}$and the proof of the main result.**

Model (2.1) has a disease-free periodic solution $E_{0}=(0,0)$. To estimate the basic reproduction ratio $R_{0}$ of model (2.1), according to the general procedure established by Wang and Zhao [[[1]](#endnote-2)], we first introduce two $2\times2$ matrices,

$$F(t)=\left(\begin{matrix} 0 & a(t)\Delta\\ 0 & 0 \end{matrix}\right),$$

$$V(t)=\left(\begin{matrix} g & 0 \\ -b(t)\frac{\Sigma}{\Delta} & \mu\end{matrix}\right).$$

It is easy to see that $F(t)$ is non-negative, and $-V(t)$ is cooperative in the sense that the off-diagonal elements of $-V(t)$ are non-negative. Let $\Phi_{V}(t)$ and be the monodromy matrix of the linear $\omega$-periodic system $\frac{dz}{dt}=V(t)z$ and the spectral radius of $\Phi_{V}(\omega)$, respectively. For the linear $\omega$-periodic system,

$\frac{dy}{dt}=-V(t)y$, (3.1)

assume that $Y(t,s),t\geq s$, is the evolution operator of this system. That is, for each $s\in R$, the $2\times2$ matrix $Y(t,s)$ satisfies

$$\frac{d}{dt}Y(t,s)=-V(t)Y(t,s),\forall t\geq s,Y(s,s)=I,$$

where *I* is the $2\times2$ identity matrix. Thus, the monodromy matrix $\Phi_{-V}(t)$ of (3.1) is equal to $Y(t,0)$, $t\geq0$.

In view of the periodic environment, we assume that , $\omega$-periodic in *s*, is the initial distribution of infectious individuals. Then is the rate of new infections produced by the infected individuals who were introduced at time *s*. Given $t\geq s$, then gives the distribution of those infected individuals who were newly infected at time *s* and remain in the infected compartments at time *t*. It follows that

is the distribution of accumulative new infections at time *t* produced by all those infected individuals introduced at time previous to *t*.

Let $C_{\omega}$ be the ordered Banach space of all $\omega$-periodic functions from *R* to *R*^2^, which is equipped with the maximum norm and the positive cone . Then we can define a linear operator $L:C_{\omega}\to C_{\omega}$ by

where *L* is called the next infection operator. The basic reproduction ratio of system (2.1) is defined as the spectral radius of *L*, i.e. $R_{0}=\rho(L)$.

In order to characterize$R_{0}$, we consider the following linear $\omega$-periodic equation

$\frac{dw}{dt}=\left[-V(t)+\frac{F(t)}{\lambda}\right]w, t\in R,$ (3.2)

with parameter $\lambda\in(0,\infty)$. Let $W(t,s,\lambda),t\geq s,s\in R$, be the evolution operator of the system (3.2) on *R*^2^. We have

$$\begin{matrix} W(\omega,0,\lambda)=exp\left[\int_{0}^{\omega} \left(-V(t)+\frac{F(t)}{\lambda}\right)dt\right] \\ =exp\left[\int_{0}^{\omega} \left(\begin{matrix} -g & \frac{a(t)\Delta}{\lambda} \\ \frac{b(t)\Sigma}{\Delta} & -\mu\end{matrix}\right)dt\right], \forall\lambda>0. \end{matrix}$$

It is easy to verify that system (2.1) satisfies assumptions (A1)-(A7) in [Error: Reference source not found]. Thus, we have the following two results, which will be used in our numerical computation of the basic reproduction ratio and the proof of our main result, respectively.

**Lemma 3.1**. The following statements are valid:

(i) If $\rho(W(\omega,0,\lambda))=1$ has a positive solution $\lambda_{0}$, then $\lambda_{0}$ is an eigenvalue of *L*, and hence $R_{0}>0$.

(ii) If $R_{0}>0$, then $\lambda=R_{0}$ is the unique solution of $\rho(W(\omega,0,\lambda))=1$.

(iii) $R_{0}=0$ if and only if $\rho(W(\omega,0,\lambda))<1$ for all $\lambda>0$.

**Lemma 3.2**. The following statements are valid:

(i) $R_{0}=1$ if and only if $\rho(\Phi_{F-V}(\omega))=1$.

(ii) $R_{0}>1$ if and only if $\rho(\Phi_{F-V}(\omega))>1$.

(iii) $R_{0}<1$ if and only if $\rho(\Phi_{F-V}(\omega))<1$.

Thus, the disease-free equilibrium $E_{0}$ is locally asymptotically stable if $R_{0}<1$, and unstable if $R_{0}>1$.

By Lemma 3.1(ii), we know that the basic reproduction ratio is determined by parameter $\lambda$ of $\rho(W(\omega,0,\lambda))=1$. We can calculate the basic reproduction ratio $R_{0}$ using the numerical method.

In the autonomous case, i.e. $a(t)\equiv a$ and $b(t)\equiv b$ for any $t\geq0$, we obtain $F(t)\equiv F$, and $V(t)\equiv V$ for any $t\geq0$, the basic reproduction ratio of the disease is $R'_{0}=ab\Sigma/(g\mu),$which corresponds to the result of Barbour (1996) [[[2]](#endnote-3)].

In the following, we prove the main result, which shows that $R_{0}$ is a threshold parameter for the extinction and the uniform persistence of the schistosomiasis model (2.1).

We first define $X:=\{(P,y):0\leq P\leq1,0\leq y\leq1\}$ for model (2.1). It is easy to prove the following theorem.

**Theorem 3.1.** Model (2.1) has a unique solution with the initial value

$(P^{0},y^{0})\in X:=\{(P,y):0\leq P\leq1,0\leq y\leq1\}$,

and this compact set is positively invariant.

Define

$X_{0}:=\{(P,y)\in X:P>0,y>0\}, \partial X_{0}:=X/X_{0}$.

Let $H:X\to X$ be the Poincare map associated with model (2.1), that is,

$H(x^{0})=u(\omega,x^{0}), \forall x^{0}\in X$,

where $u(t,x^{0})$ is the unique solution of model (2.1) with $u(0,x^{0})=x^{0}$. It is easy to see that

$H^{m}(P^{0},y^{0})=u(m\omega,(P^{0},y^{0})), \forall m\geq0$.

We establish the following lemma which will be useful in subsequent main result.

**Lemma 3.3.**  If the basic reproduction ratio $R_{0}>1$, then there exists a $\sigma^{*}>0$, such that for any $(P^{0},y^{0})\in X_{0}$ with $\left\|(P^{0},y^{0})-E_{0}\right\|\leq\sigma^{*}$, we have

$\limsup_{m\to\infty} d(H^{m}(P^{0},y^{0}), E_{0})\geq\sigma^{*}.$ (3.3)

**Proof.** Since $R_{0}>1$, Lemma 3.2 implies $\rho(\Phi_{F-V}(\omega))>1$. It follows that $\rho(\Phi_{F-V-M_{\sigma}}(\omega))>1$ holds for sufficiently small $\sigma>0$, where

$$M_{\sigma}=\left(\begin{matrix} 0 & a(t)\Delta\sigma\\ b(t)\frac{\Sigma}{\Delta}\sigma& 0 \end{matrix}\right).$$

By the continuity of the solutions with respect to the initial values, there exists a $\sigma^{*}>0$ such that for all $(P^{0},y^{0})\in X_{0}$ with $\left\|(P^{0},y^{0})-E_{0}\right\|\leq\sigma^{*}$, there holds $\left\|u(t,(P^{0},y^{0}))-u(t,E_{0})\right\|\leq\sigma$, for all $t\in[0,\omega]$. Next, we claim that $\limsup_{m\to\infty} d(H^{m}(P^{0},y^{0}), E_{0})\geq\sigma^{*}$. Assume, by contradiction, that (3.3) does not hold. Then we have

$$\limsup_{m\to\infty} d(H^{m}(P^{0},y^{0}), E_{0})<\sigma^{*}$$

for some $(P^{0},y^{0})\in X_{0}$. Without loss of generality, we assume that $d(H^{m}(P^{0},y^{0}), E_{0})<\sigma^{*}$, for all $m\geq0$. It follows that

$\left\|u(t,H^{m}(P^{0},y^{0}))-u(t,E_{0})\right\|=\left\|u(t,H^{m}(P^{0},y^{0}))\right\|<\sigma, \forall m\geq0, \forall t\in[0,\omega]$.

For any $t\geq0$, let $t=m\omega+t'$, where $t'\in[0,\omega)$, and $m$ is the largest integer less than or equal to $t/\omega$. Therefore, we have

$\left\|u(t,(P^{0},y^{0})-u(t,E_{0}))\right\|=\left\|u(t',H^{m}(P^{0},y^{0}))\right\|<\sigma, \forall t\geq0$.

Note that $(P(t),y(t))=u(t,(P^{0},y^{0}))$. It then follows that $P(t)<\sigma$, $y(t)<\sigma$, for all $t\geq0$. From model (2.1), we obtain

$\left\{\begin{matrix} \frac{dP}{dt}\geq a(t)\Delta y(1-\sigma)-gP, \\ \frac{dy}{dt}\geq b(t)(\frac{\Sigma}{\Delta})P(1-\sigma)-\mu y. \end{matrix}\right.$ (3.4)

We then consider the following system

$\left\{\begin{matrix} \frac{d\hat{P}}{dt}=a(t)\Delta\hat{y}(1-\sigma)-g\hat{P}, \\ \frac{d\hat{y}}{dt}=b(t)(\frac{\Sigma}{\Delta})\hat{P}(1-\sigma)-\mu\hat{y}. \end{matrix}\right.$ (3.5)

By Zhang and Zhao ([[[3]](#endnote-4)], Lemma 2.1), we know that there exists a positive, $\omega$-periodic function $(\bar{P}(t),\bar{y}(t))^{T}$ such that $(\hat{P}(t),\hat{y}(t))^{T}=e^{\xi t}(\bar{P}(t),\bar{y}(t))^{T}$ is a solution of system (3.5), where$\xi=\frac{1}{\omega}ln\rho(\Phi_{F-V-M_{\sigma}}(\omega))$. Since $\rho(\Phi_{F-V-M_{\sigma}}(\omega))>1$, $\xi$ is a positive constant. Let $t=n\omega$, and $n$ be any nonnegative integer, and we get

$$(\hat{P}(n\omega),\hat{y}(n\omega))^{T}=e^{\xi n\omega}(\bar{P}(n\omega),\bar{y}(n\omega))^{T}\to(\infty,\infty)^{T}$$

as $n\to\infty$, since $\omega\xi>0$ and $(\bar{P}(t),\bar{y}(t))^{T}>0$. For any nonnegative initial condition $(P(0),y(0))^{T}$ of system (3.4), there exists a sufficiently small $m^{*}>0$ such that $(P(0),y(0))^{T}\geq m^{*}(\bar{P}(0),\bar{y}(0))^{T}$. By the comparison principle ([[[4]](#endnote-5)], Theorem B.1), we have $(P(t),y(t))^{T}\geq m(\hat{P}(t),\hat{y}(t))^{T}$, for all $t>0$. Thus, we obtain $P(n\omega)\to\infty$ and $y(n\omega)\to\infty$, as $(n\to\infty)$, a contradiction.

Then we have the following Theorem.

**Theorem 3.2.** If the basic reproduction ratio $R_{0}<1$, then the unique disease-free equilibrium $E_{0}=(0,0)$ is globally asymptotically stable. If the basic reproduction ratio $R_{0}>1$, then there exists a constant $\delta>0$ such that any solution $(P(t),y(t))$ of system (2.1) with initial value

$(P(0),y(0))\in X_{0}$($\text{X}_{0}\text{:=\{(P,y)}\in\text{X : P>0, y>0\}}$)

satisfies

$\liminf_{t\to\infty} P(t)\geq\delta$ and $\liminf_{t\to\infty} y(t)\geq\delta$.

**Proof:** By Lemma 3.2, we know that if $R_{0}<1$, then $E_{0}$ is locally asymptotically stable. It is sufficient to prove that $E_{0}$ is globally attractive if $R_{0}<1$. From system (2.1), we have

$\left\{\begin{matrix} \frac{dP}{dt}\leq a(t)\Delta y-gP, \\ \frac{dy}{dt}\leq b(t)(\frac{\Sigma}{\Delta})P-\mu y. \end{matrix}\right.$ (3.6)

Consider the following comparison system

$\frac{dh(t)}{dt}=(F(t)-V(t))h(t).$ (3.7)

Applying Lemma 3.2, we know that $R_{0}<1$ if and only if $\rho(\Phi_{F-V}(\omega))<1$. By Zhang and Zhao ([Error: Reference source not found], Lemma 2.1), it follows that there exists a positive, $\omega$-periodic function $\bar{h}(t)$ such that $h(t)=e^{\theta t}\bar{h}(t)$ is a solution of system (3.7), where $\theta=\frac{1}{\omega}ln\rho(\Phi_{F-V}(\omega))$. Since $\rho(\Phi_{F-V}(\omega))<1$, $\theta$ is a negative constant. Therefore, we have $h(t)\to0$ as $t\to+\infty$. This implies that the zero solution of system (3.7) is globally asymptotically stable. For any nonnegative initial value $(P(0),y(0))^{T}$ of system (3.6), there is a sufficiently large $M^{*}>0$ such that $(P(0),y(0))^{T}\leq M^{*}\bar{h}(0)$ holds. Applying the comparison principle ([Error: Reference source not found], Theorem B.1), we have $(P(t),y(t))^{T}\leq M^{*}h(t)$, for all $t>0$, where $M^{*}h(t)$ is also the solution of system (3.7). Therefore, we get $P(t)\to0$, and $y(t)\to0$ as $t\to0$. We finish the proof of the first part of the theorem.

By Theorem 3.1, the discrete-time system $\{H^{m}\}_{m\geq0}$ admits a global attractor in $X$. Now we prove that $\{H^{m}\}_{m\geq0}$ is uniformly persistent with respect to $(X_{0},\partial X_{0})$. Clearly, there is exactly one fixed point $E_{0}=(0,0)$ of $H$ in $\partial X_{0}$. Lemma 3.3 implies that $E_{0}=(0,0)$ is an isolated invariant set in $X$ and $W^{s}(E_{0})\bigcap X_{0}=\emptyset$. By Zhao ([[[5]](#endnote-6)], Theorem 1.3.1), it follows that $H$ is uniformly persistent with respect to $(X_{0},\partial X_{0})$. By Zhao ([Error: Reference source not found], Theorem 3.1.1), the solutions of system (2.1) are uniformly persistent with respect to $(X_{0},\partial X_{0})$, that is, there exists a constant $\delta>0$ such that any solution $(P(t),y(t))$ of system (2.1) with initial value $(P(0),y(0))\in X_{0}$ satisfies

$\liminf_{t\to\infty} P(t)\geq\delta$ and $\liminf_{t\to\infty} y(t)\geq\delta$.

1. [?] Wang WD, Zhao XQ: **Threshold dynamics for compartmental epidemic models in periodic environments**. *J Dyn Diff Equat* 2008, **20**:699-717. [↑](#endnote-ref-2)
2. [?] Barbour AD: **Modelling the transmission of schistosomiasis an introductory view.** *Am J Trop Med Hyg* 1996, **55** (5 Suppl): 135-143. [↑](#endnote-ref-3)
3. [?] Zhang F, Zhao XQ: **A periodic epidemic model in a patchy environment.** *J Math Anal Appl* 2007, **325**: 496-516. [↑](#endnote-ref-4)
4. [?] Smith HL, Walman P: **The Theory of the Chemostat.** *Cambridge University Press,* Cambridge, 1995. [↑](#endnote-ref-5)
5. [?] Zhao XQ: **Dynamical Systems in Population Biology.** *Springer,* New York 2003. [↑](#endnote-ref-6)
